# “Everything has been tried and his heart can’t recover…”: A Descriptive Review of “Do Everything!” in the Archive of Ontario Consent and Capacity Board

**DOI:** 10.1186/s12910-022-00796-7

**Published:** 2022-06-27

**Authors:** Holly Yim, Syeda Shanza Hashmi, Brian Dewar, Claire Dyason, Kwadwo Kyeremanteng, Susan Lamb, Michel Shamy

**Affiliations:** 1 Department of Medicine, University of Ottawa, 1053 Carling Avenue, Ottawa, ON Canada; 2 Department of Psychiatry, University of Toronto, 250 College Street, Toronto, ON Canada; 3 Ottawa Hospital Research Institute, 1053 Carling Avenue, Ottawa, ON Canada; 4 Department of Innovation in Medical Education, Jason A. Hannah Chair in History of Medicine, University of Ottawa, 451 Smyth Road, Ottawa, ON Canada

**Keywords:** Communication, Bioethics, Palliative care, Goals of care, Critical care

## Abstract

**Background:**

In end-of-life situations, the phrase “do everything” is sometimes invoked by physicians, patients, or substitute decision-makers (SDM), though its meaning is ambiguous. We examined instances of the phrase “do everything” in the archive of the Ontario Consent and Capacity Board (CCB) in Canada, a tribunal with judicial authority to adjudicate physician–patient conflicts in order to explore its potential meanings.

**Methods:**

We systematically searched the CCB’s online public archive from its inception to 2018 for any references to “do everything” in the context of critical care medicine and end-of-life care. Two independent assessors reviewed decisions, collected characteristics, and identified key themes.

**Results:**

Of 598 cases in the archive, 41 referred to “do everything” in end-of-life situations. The phrase was overwhelmingly invoked by SDMs (38/41, 93%), typically to advocate for life-prolonging measures that contradicted physician advice. Physicians generally related “doing everything” to describe the interventions they had already performed (3/41, 7%), using it to recommend focusing on patients’ quality of life. SDMs were generally reluctant to accept death, whereas physicians found prolonging life at all costs to be morally distressing. The CCB did not interpret appeals to “do everything” legally but followed existing laws by deferring to patients’ prior wishes whenever known, or to concepts of “best interests” when not. The CCB generally recommended against life-prolonging measures in these cases (26/41, 63%), focusing on patients’ “well-being” and “best interests.”

**Conclusions:**

In this unique sample of cases involving conflict surrounding resuscitation and end-of-life care, references to “do everything” highlighted conflicts over quantity versus quality of life. These appeals were associated with signs of cognitive distress on the behalf of SDMs who were facing the prospect of a patient’s death, whereas physicians identified moral distress related to the prolongation of patients’ suffering through their use of life-sustaining interventions. This divergence in perspectives on death versus suffering was consistently the locus of conflict. These findings support the importance of tools such as the Serious Illness Conversation Guide that can be used by physicians to direct conversations on the patients’ goals, wishes, trade-offs, and to recommend a treatment plan that may include palliative care.

***Trial Registration*:**

Not applicable.

## Background

Regrettably, conflicts surrounding end-of-life decisions may arise at the bedsides of critically ill patients in up to 80% of cases [1] and have been prominently featured in both the medical literature [2] and the popular press [3]. Legal challenges have arisen surrounding prominent cases such as that of the Terri Schiavo [4] in the United States (1998–2005) and Cuthbertson v. Rasouli in Canada, which reached the Supreme Court in 2013 [5]. The current COVID-19 pandemic has seemingly accentuated the longstanding tension surrounding end-of-life decisions, fundamental values, and ethical principles that arises around the application of critical care in catastrophic cases [6, 7].

In the course of such conflicts, the phrase “do everything” may be introduced by physicians, patients, or substitute decision-makers (SDM) [8]. For example, some physicians may ask patients whether they would want to “do everything” for resuscitation, offering a “resuscitation menu” [9] of options such as cardiopulmonary resuscitation and mechanical intubation. This approach may unknowingly create a false impression that these interventions would be appropriate and successful [10, 11]. Alternatively, patients’ substitute decision-makers may request that physicians “do everything”, though they may not envision interventions such as tracheostomy, central venous catheter insertion, dialysis and vasopressor use [12]. Some studies suggest that patients are less likely to accept a physician’s recommendation to withdraw life-sustaining treatment when this is contrasted with “doing everything” [13], perhaps due to the perception that the patient will receive inferior care [11]. Whether expressed as a directive to physicians, or as an assurance by physicians to patients or their loved ones, the meaning of “do everything” remains ambiguous and threatens to obscure uncomfortable choices faced by physicians, patients, and their families [14] .

This study analyzes the meaning of “do everything” by examining its use in the archive of the Consent and Capacity Board (CCB) of Ontario in Canada. The CCB is an independent tribunal made up of lawyers, physicians, nurses, and community members appointed by the province’s Lieutenant Governor [15, 16]. It was established under the provincial *Health Care Consent Act* (HCCA) to adjudicate disputes surrounding substitute decision-making in a fair and unbiased manner. In order for cases to reach the CCB, patients have to be incapable such that they do not understand relevant information for decision-making and are unable to appreciate the foreseeable consequences of their decision or lack of decision. Therefore, relevant parties may include the patient’s authorized substitute decision-makers (SDM), other stakeholders, and the medical team. Both the medical team or a patient’s SDM can apply to the CCB if an intractable conflict arises [17]. The CCB can direct the SDM to consent to part or all of the treatment plan proposed by physicians, or may direct the medical team to comply with SDMs’ requests [17]. Any party involved in the case may appeal the CCB’s decision to the Superior Court of Justice of Ontario [15]. Final decisions of the CCB are rendered by lawyers with expertise in health law and medical ethics. CCB decisions are presented in a narrative format that summarizes the participants’ testimony and include direct quotations from petitioning parties.

The objective of this study is to explore the meaning of the phrase “do everything” as captured within the decisions of the CCB, and to characterize the contexts in which it was invoked. These CCB decisions represent real-life scenarios in which conflicts surrounding substitute-decision making were unresolved through local hospital processes, therefore necessitating legal intervention. Short of being present for conversations between physicians and patient’s SDMs, the archive of the CCB provides the most transparent window to examine and understand the complex conflicts that can arise surrounding end-of-life decision-making. Ultimately, we hope that better understanding of appeals to “do everything” will improve the oftentimes challenging communication that occurs in the care of critically ill patients and patients at the end-of-life.

## Methods

**Fig. 1:**
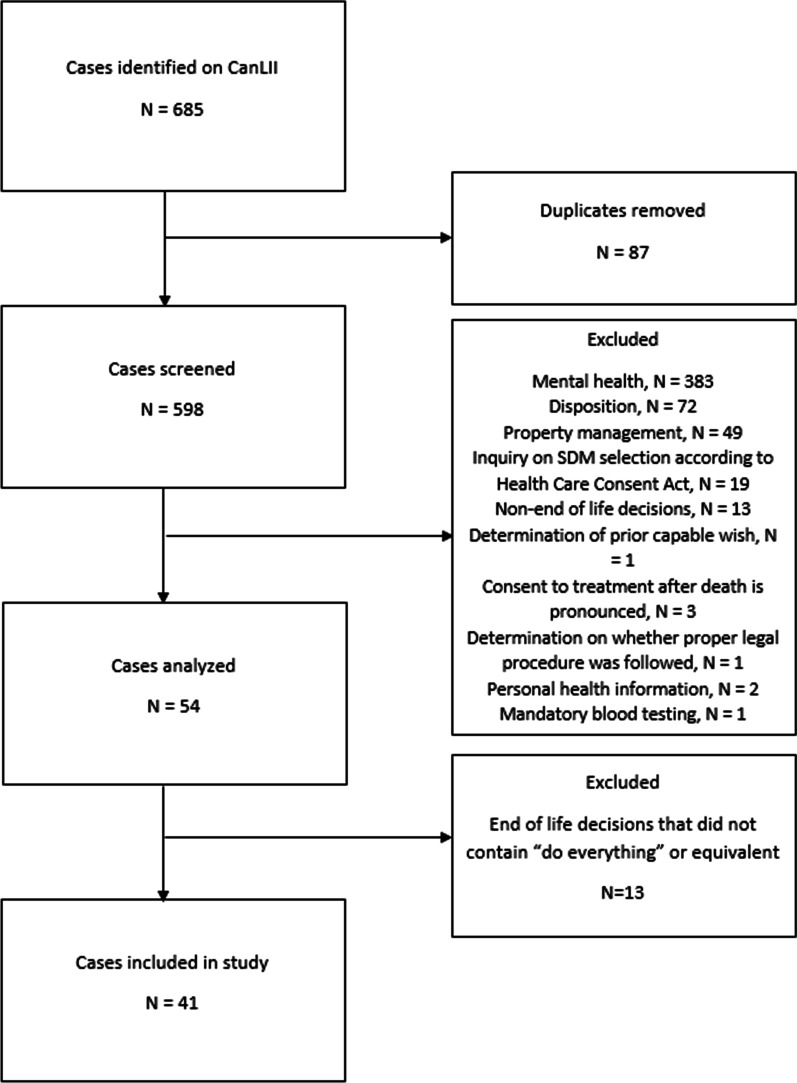
Figure 1 details the inclusion and exclusion criteria. 598 cases were screened. Common reasons for exclusion were mental health, disposition determination, and property management. 41 cases met inclusion criteria and were included in the analysis of the study

We systematically searched all cases in the CCB archive from its inception in 2003 to 2018 on the publicly accessible Canadian Legal Information Institute database (CanLII) [18] for any references to the phrase “do everything” or related phrases in the context of critical care medicine. Figure 1 presents the screening process, inclusion, and exclusion criteria. Two independent reviewers (HY, SSH) analyzed all decisions and collected characteristics of each decision. They compared their extractions and resolved any disagreements by consensus. A third reviewer (MS) adjudicated any discrepancies that could not be resolved by the two primary reviewers.

These two reviewers also extracted any text excerpts that contained “do everything” or related phrases. Text excerpts included direct quotations or statements from patients, SDMs, or physicians present during the hearing and quotations from clinical documents submitted as evidence. Once text excerpts were identified, reviewers used Braun and Clarke’s Thematic Analysis [19] to code and generate key themes from the extracted quotations. They reached a consensus on all codes and themes and interpreted the themes to understand the context and meaning of “do everything”.

## Results

**Fig. 2:**
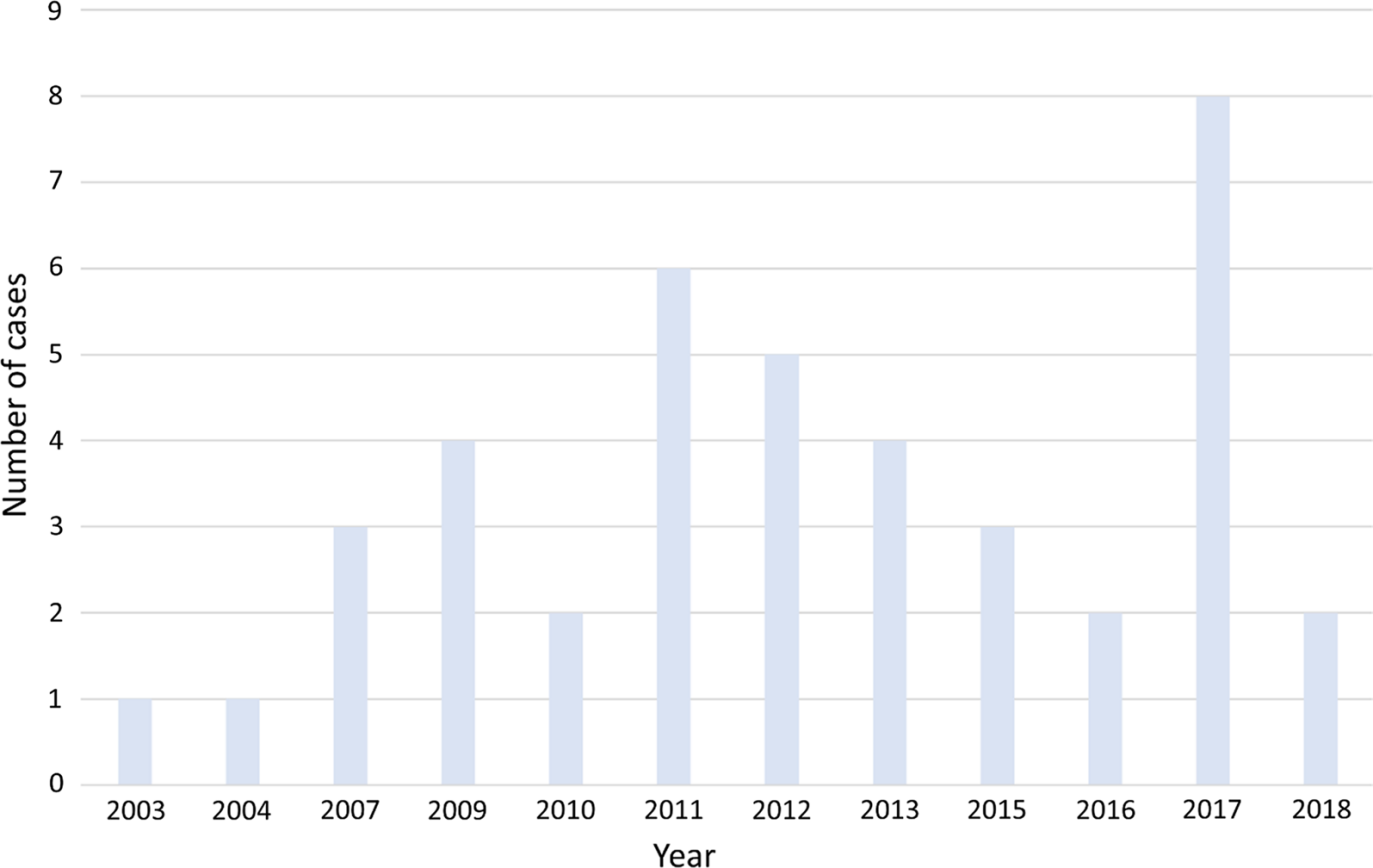
Figure 2 details the year-by-year breakdown of cases. References to “do everything” related to end-of-life decision-making appear to be becoming more common, with nearly half the cases (19/41) occurring in the last 5 years and the single highest year being 2017

*Frequency of use:* Of 598 cases in the CCB archive, 41 (6.9%) included the phrase “do everything” or related phrases in end-of-life situations. Most cases (33/41, 80%) occurred in a critical care setting and few (3/41, 7%) occurred in an acute care setting (Table 1). The phrase “do everything” also appeared in cases related to the Mental Health Act, but these were not specifically analyzed according to our inclusion criteria. Examples of excluded cases included those in which a patient’s status as an incapable person was reviewed in relation to their psychiatric disorder or the determination of the facility that an incapable person would be discharged to, which is labelled as “disposition” (Fig. 1). References to “do everything” related to end-of-life decision-making appeared to be becoming more common, with nearly half the cases (19/41) occurring in the last 5 years and the single highest year being 2017 (Fig. 2).

**Table 1:** Summary of characteristics of all cases

Description	N	%
Total included cases	41	
*Setting*		
Critical Care	33	80.5
Acute Medicine	3	7.3
Pediatric critical care	2	4.9
Unable to determine	3	7.3
*Conflict over*		
Withdrawing life-sustaining therapy	31	75.6
Withholding life-sustaining therapy	9	22.0
Did not specify	1	2.4
*Patient condition*		
Altered level of consciousness	25	61.0
Terminal illness	16	39.0
*Diagnoses*		
Dementia	13	31.7
Anoxic / hypoxic brain injury	11	26.8
Stroke	10	24.4
Cardiac Arrest	8	19.5
Infection	15	36.6
Kidney failure	7	17.1
Multi-organ failure	6	14.6
GI bleed	4	9.8
Pressure ulcers	6	14.6

Table 1 provides a summary of characteristics of all cases. In total, 41 cases were included in the study. Most cases (80.5%) took place in a critical care setting where most conflicts (75.6%) were related to withdrawal of life-sustaining therapy. Patients typically had more than one diagnosis, with leading diagnoses being infection (36.6%), dementia (31.7%), and anoxic or hypoxic brain injury (26.8%)

*Context:* The phrase “do everything” appeared overwhelmingly in relation to statements made by SDMs (38/41, 93%), rather than by the medical team (3/41, 7%). In these 38 cases in which “do everything” was invoked by the SDMs, 28 cases (74%) involved conflicts around withdrawal of life-sustaining therapy, such as removal of mechanical ventilation, or dialysis, while nine cases (24%) involved withholding life-sustaining therapy (i.e., not offering therapy). In one case, the topic of dispute was not described. Patient characteristics and diagnoses are included in Table 1.

SDMs opposed the medical team’s opinion and appealed for life-sustaining intervention in 34/38 (89%) cases in which they invoked "do everything." In the remaining four cases, one involved the SDM appealing to withhold blood transfusion for religious reasons and the other three cases reflected disagreement among SDMs about the course of action to be taken. In the three cases where the medical team used “do everything”, it was always in reference to having already done everything that could be done without a positive outcome for the patient. The medical teams’ statements around having done everything served as the basis to propose a treatment plan focusing on patient comfort, such as withdrawing life-sustaining measures.

*Decisions:* The CCB deferred to patients’ prior expressed and applicable wishes whenever known, as in 11 cases. Only 3/11 (27%) patients had expressed a wish to have life-sustaining interventions, yet all 11 SDMs advocated for life-sustaining interventions. Accordingly, the CCB ruled for life-sustaining interventions in those three cases and against life-sustaining interventions in the remaining eight cases. In the absence of prior capable wishes (30 cases), the CCB appealed to the concept of “well-being” and tended to recommend against life-sustaining interventions (18/30, 60%). They ruled for providing or continuing life-sustaining interventions in 7 cases (23%) and declined to render a decision in 5 cases (17%).

The CCB supported the medical team’s recommendation to withdraw life-sustaining interventions in all three cases in which the medical team used the phrase “do everything.” The CCB did not offer a specific legal interpretation of “do everything” in their decisions. Instead, they followed the wording of Ontario’s Health Care Consent Act to analyze cases with reference to the legal definition of “best interest” (Table 2), paying specific attention to the concept of “well-being” within that legal definition. The CCB provided specific recommendations on individual interventions, including “palliation” and “comfort measures” in 25/41 (61%) cases.

**Table 2:** The legal definition of “Best Interest”

**“Best Interest” definition**^**32**^ (2) In deciding what the incapable person’s best interests are, the person who gives or refuses consent on his or her behalf shall take into consideration, (a) the values and beliefs that the person knows the incapable person held when capable and believes he or she would still act on if capable; (b) any wishes expressed by the incapable person with respect to the treatment that are not required to be followed under paragraph 1 of subsection (1); and (c) the following factors: 1. Whether the treatment is likely to, i. improve the incapable person’s condition or well-being, ii. prevent the incapable person’s condition or well-being from deteriorating, or iii. reduce the extent to which, or the rate at which, the incapable person’s condition or well-being is likely to deteriorate 2. Whether the incapable person’s condition or well-being is likely to improve, remain the same or deteriorate without the treatment 3. Whether the benefit the incapable person is expected to obtain from the treatment outweighs the risk of harm to him or her 4. Whether a less restrictive or less intrusive treatment would be as beneficial as the treatment that is proposed. 1996, c. 2, Sched. A, s. 21 (2)	

Table 2 shows the definition of “Best Interest” as defined in the Health Care Consent Act

*Key Themes:* Thematic analysis of these 41 cases led to the differentiation of three key themes: quantity of life versus quality of life, cognitive distress related to the prospect of the patient’s death, and moral distress related to the patient’s suffering.

SDMs invoked “do everything” to appeal for interventions that would prolong the patient’s life. These appeals were frequently based on their understanding of patients’ wishes to live (23/38, 61%), beliefs in religion or faith (12/38, 32%), or hope (8/38, 21%). SDMs used “do everything” as a medical imperative with phrases such as “do everything possible” [20], “do everything to preserve [life]” [21], “everything had to be done to continue life” [22] (Table 3). In contrast, the medical team advocated for treatment plans that would prioritize the patient’s quality of life. They used “do everything” to encapsulate the various interventions that had already been undertaken that had failed to improve the patient’s condition. One physician stated that “everything has been tried and that his heart can’t recover” [23]. The medical team recommended focusing on the patient’s quality of life by withdrawing life-sustaining interventions. One physician made this trade off explicit when they stated that “death would alleviate [the patient’s] suffering and improve [the patient’s] well-being” [23] (Table 3).

**Table 3:** Sample quotations illustrating the different ways that “Do Everything” were used by substitute decision-makers and medical team

“Do Everything” as used by SDMs (Focusing on Quantity of Life)	“Do Everything” as used by medical team (Focusing on Quality of Life)
*“Do everything possible”* “[SDM] refused consent to withdrawal of life support, insisting that the medical team continue to **do everything possible** to extend [patient’s] life… [Patient] would often say things like “no matter what, you keep me alive, I want CPR, whatever it takes – you’d better remember!”, “no matter how many tubes or needles, to the nth degree, I still want it.”^20^ “SDM said his father (patient) would have wanted **everything done that was possible** to do because he was a fighter… SDM said [patient’s] motto was “where there is life, there is hope” …”^25^	*“Patient is dying despite having tried everything available”*
	“In my and many other health care providers’ opinion [patient] is dying and we are proposing palliative care. We have **exhausted all medical treatment** (aggressive and routine) available to us. Further aggressive care would pose more risk than benefit. [SDM] do not understand that [patient’s] condition is irreversible and terminal and that **[the medical team] have tried everything available** such as life support via a ventilator, antibiotics, artificial feeding and despite this **his overall health continues to decline.** [SDM] believe that further feeding, admission to ICU, and medication will make [patient] regain his strength and cognitive function when only the opposite is true… A plan of treatment that was focused on palliative care and would improve [patient’s] well being. **Although it could shorten [patient’s] life, it would ensure that he was kept comfortable and would improve his quality of life and respect his dignity**.”^24^ “Initially with [doctor’s name] and then with [doctor’s name] to provide further confirmation to family that **everything has** **been tried and that his heart can’t** **recover…** [Doctor] considered the current course of treatment to be a form of torture for [patient]…that his patient was suffering while on the life support machines. [Doctor] recommended the proposed course of treatment [palliative care] as a way to allow his patient to lessen his pain and to die in dignity… [Patient] would likely pass away within a short time of removal of the life support machines. **His death would alleviate [patient’s] pain and suffering and improve [patient’s] well-being in the doctor’s opinion.”**^23^
*“Preserving” life*	
“As a practicing Catholic, father (patient)	
believes that life is a gift given by God and it	
is a duty to **do everything to preserve it.**”^21^ *“Continuing” life* “SDM said she and [patient] talked about life support and [patient’s] view was that you did everything you could to fight. **Everything** **had to be done to continue life…** He would want to stay alive as long as possible… to endure pain in exchange for the joy of seeing his family present at his bedside”^22^ *“Prolonging” life* “[SDM] had hopes and they wanted [patient] **“continuously to be treated.”** In [SDM]’s impassioned evidence, she said that the **doctors’ job was to prolong life.** She said that her father was not living on machines alone… because he was “responsive to us, we feel certain diagnoses were wrong.” She said that doctors were human and they made mistakes.”^26^	
	*“Without providing everything SDM* *wanted”* “The doctor also said the focus of the proposed plan [including palliative care] was [patient]’s best interest but **without providing everything that the family wanted** and believed was in [patient]’s best interests.”^42^

Table 3 shows sample quotations illustrating the different ways that “Do Everything” were used by substitute decision-makers and medical team. The substitute decision-makers tended to focus on quantity of life where the medical team would focus on quality of life. In general, “do everything” was invoked by substitute decision-makers to prolong or continue life at all costs, i.e., to “do everything possible”. In contrast, the medical team invoked “do everything” to reflect the various forms of interventions that had already been undertaken that failed to improve the patients’ health, i.e., “everything has been tried and that his heart can’t recover.” Specific words were bolded for emphasis as related to the theme

Second, SDMs appeared to have experienced significant cognitive distress around the prospect of accepting the patient’s death (Table 4). SDMs expressed mistrust of the medical team, guilt at having to make decisions, and a desire not to be responsible for the consequences of those decisions. Two SDMs felt that pursuing palliation was akin to euthanasia, and seven expressed the belief that prolonging a patient’s life was more important than relieving suffering.

**Table 4:** Sample quotations illustrating the distress that substitute decision-makers and medical team experienced

Cognitive distress experienced by SDMs	Moral distress experienced by Medical Team
*Mistrust of Medical Team* “In [SDM]’s evidence, they variously insisted [patient] was not **“that sick, he’s only having trouble breathing,”** that he **would want everything done**, that he wanted to die at home.”^43^ “[SDM] had hopes and they wanted [patient] **“continuously to be treated.”** In [SDM]’s impassioned evidence, she said that the **doctors’ job was to prolong life.** She said that her father was not living on machines alone. She said that because he was “responsive to us**, we feel certain diagnoses were wrong**.” She said that doctors were human and they made mistakes.”^26^	“[Physician] testified that [SDMs] were insisting on Full Code status because they were respecting [patient’s] wishes, values and beliefs. However, [physician] said, **these were now impossible to respect because they violated physician ethical principles**… Patients could not dictate treatment, that full treatment here was medically futile, not medically effective that [patient] would die regardless. **[Physician] could not be required to act outside of the standard of care for [patient]. [Physician] believed that what he was being asked to provide, by following [patient]’s wishes was morally and ethically at odds with his physician’s oath to do no harm.”**^21^ “[Physician] stated his belief that if [patient] could speak now, he would not want to remain on life support because **he was suffering inhumanely with no potential benefits from treatment.** [Physician] said that [patient] was in the process of dying, and they **[SDMs] were only prolonging his death**, rather than providing any meaningful life. [Physician] felt that **nobody would ever want to prolong their dying days in this manner.”**^20^ “[Physician] stated that there was a difference between prolonging life and “living” and, at this point, aggressive medical intervention was only prolonging [patient’s] life and increasing his suffering… It was his opinion that **no one would want to continue life in that manner given that “this was not a pleasant way to exist”** and that there was no chance of “meaningful recovery.””^44^ “In [physician’s] opinion the focus should be on quality of [patient’s] life, not duration. [Patient’s] well-being should focus on comfort, that **life-support was uncomfortable and would serve to prolong [patient’s] dying and add to his suffering.”**^25^ “[Physician] proposed a plan of treatment that was focused on palliative care and would improve his [patient’s] well being. Although it could shorten his life, it would ensure that he was kept comfortable and would improve his quality of life and respect his dignity. **[Physician] told the Board that [patient] felt pain and was uncomfortable when the numerous life preserving procedures were being performed.”**^24^
*Guilt* “[SDM] **wanted everything done** for [patient]. He said: “I won't change my mind. I don't want to feel **guilty**. I don't want to kill my blood.””^45^	
*Acceptance of Tradeoff between Quality and Quantity of Life* “**[SDM] did not think [patient] would want to suffer to stay alive but also said [the medical team] should do everything** they could for [patient], including resuscitation because it was **better to be alive.**”^43^ “[SDM] said that [doctor] was correct that [patient] needed many tubes and she understood that the treatment team thought that these were uncomfortable for [patient]. However, she stated that [patient] would prefer to be uncomfortable, **even in the face of the dementia and his dependence, that he would choose discomfort over a decision to stop taking all possible steps to maintain his life.”**^44^	

Table 4 shows sample quotations illustrating the cognitive distress and moral distress that substitute decision-makers and medical teams experienced, respectively. In invoking “do everything”, some substitute decision-makers discussed their mistrust towards the medial team in that they disagreed with the medical team’s assessment of the patient’s deteriorating status. Some have reported feeling guilty and not wanting to kill the patient. Others accepted that prolonging life was worthwhile, even at the expense of the patient’s quality of life. For members of the medical team, some found pursuing life-prolonging interventions inhumane, uncomfortable for patients, and even at odds with their duty to do no harm. They appeared to be distressed that ongoing life-sustaining interventions would add to their patient’s suffering without any benefits. Specific words were bolded for emphasis as related to the theme

These positions contrasted with the positions of the medical teams, who expressed that SDMs’ request to prolong life at all costs was morally distressing for them (Table 4). One physician felt that following the SDM’s wish was “morally and ethically contradictory to his oath to do no harm” [21]. Other physicians found these life-prolonging interventions “inhumane” [20], “uncomfortable” [24, 25], and only served to add to the patient’s suffering and pain without any benefits or increasing the chance of a meaningful recovery.

## Discussion

In response to the recognized ambiguity surrounding the phrase “do everything” as it appears in the course of medical communication [14], this study demonstrates two broad conclusions regarding its uses in the CCB archive. First, references to “do everything” highlighted conflicts over quantity versus quality of life. Whereas SDMs commonly invoked the desire to “do everything” to prolong life at all costs, medical teams identified patient quality of life and likelihood of recovery as drivers of decision-making. In contrast to what one SDM in our study perceived as the physician’s role to prolong life (“the doctors’ job was to prolong life”) [26], physicians in our study objected to prolonging life at all costs and preferred palliative care when patient’s health continued to decline despite maximal medical therapy (“Although it [palliative care] could shorten [patient’s] life, it would ensure that he was kept comfortable and would improve his quality of life and respect his dignity.” [24]). SDMs were observed to continue or escalate interventions based on any small *possibility* of recovery. This was even against assurances from the medical team to the contrary, and often despite considerations of the patient's quality of life or the *probability* of success. This is illustrated in one case: “[SDM], although hopeful of a recovery, could not or refused to accept the expert doctors’ opinions that his father [patient] would not recover.” [26] The meaning of this quotation reflects that while the SDM felt that there was a *possibility* of recovery, the medical team deemed the recovery *improbable.* Decision-making based on the *possibility* of recovery can be a “tyranny of hope”, which has been described in the bioethics literature as a paradoxical effect where a focus on hope and recovery causes acceptance of physical and emotional suffering at the expense of loss of function and independence [27]. It is this divergence in perspectives on death versus suffering that was consistently the locus of conflict between SDMs and the medical team.

Second, appeals to “do everything” were associated with expressions of distress from the SDMs and the medical team, albeit of different kinds. SDMs’ statements suggest a certain cognitive distress about the prospect of the patient’s death, while the medical team expressed moral distress regarding patient suffering due to life-prolonging interventions. This once again contrasts the importance placed by the SDMs and the medical team on death versus suffering, respectively. Previous research has similarly demonstrated that SDMs perceive palliative approach as a choice between life or death, around which they express unresolved questions or even resentment towards the medical team [28, 29]. Other studies echo our results in that medical teams report “practitioner suffering” related to the treatments they offer to patients at the end-of-life, “powerlessness” in the face of SDMs’ requests for treatments they deem harmful, and the expressions of “futility” of providing care that serve not to prolong life but to prolong the dying process [30, 31].

When SDMs invoke “do everything”, how then should physicians respond? Our results suggest that emphasizing a patient’s prognosis may fail to address SDMs’ cognitive distress and may only heighten tensions, since SDMs may choose to reject the medical team’s assessments. Some physicians have appealed to the concept of “best interest” [32], but prior work has identified “best interest” as being subject to interpretation by each party involved and is unlikely to lead to consensus [33–35]. Additionally, even though “best interest” has been legally defined [32], our study demonstrated that the CCB tended to focus on the “well-being” component, which more closely aligned with concepts such as the experience of pain or suffering.

Structured tools such as the Serious Illness Conversation guide (SICG) [36] may be helpful to guide conversations between a medical team and SDMs in end-of-life circumstances. Although initially piloted with patients with advanced cancer [37], the SICG has been extended to critical care and has been shown to enhance clinicians’ understanding of patient’s wishes, SDMs’ understanding of patients’ conditions, and trust in the physician–patient relationship [38]. The SICG avoids using ambiguous phrases such as “do everything” and instead explores goals, fears, critical abilities with standardized questions such as, “what abilities are so critical to your life that you can’t imagine living without them?” and “if you become sicker, how much are you willing to go through for the possibility of gaining more time?” [36] The SICG supports SDMs to reflect on what would be the most important and necessary for the patient, rather than on prolonging life at all costs. Such reflection may help to align SDMs’ decision with the patient’s values. As our results suggest, SDMs’ desire to sustain life no matter what may often be at odds with patient’s previously expressed wishes. By answering these structured questions such as those in the SICG, SDMs may be relieved of the pressure and guilt associated with deciding not to pursue all possible interventions to sustain life. Some critical care physicians have also reported less anxiety and improved professional satisfaction when using the SICG as it facilitated discussions of realistic expectations [38]. Using the SICG can also provide an opportunity for physicians to provide recommendations on a plan of care, including recommending palliative care and explaining its active role in symptom management and affirmation of patient values. A guideline made famous by William Osler is worth repeating: our duty to patient is to cure sometimes, relieve often, and comfort always [39].

It is important to note that there is significant global variability surrounding the practice of withdrawal of life-sustaining treatment. For example, withdrawal of life-sustaining treatment is more prevalent in countries in North America and Europe than in the Middle East and Asia [40]. This may reflect the different socio-cultural values as well as political organization and economic realities. We have not identified a similar study exploring decisions around end-of-life care in a different cultural context, though such a study would reflect an important contribution to this literature.

The strengths of our study include its novelty in the use of legal transcripts to explore the meaning of phrases used in medical communication, as well as its ongoing relevance. The increasing frequency of appearances of “do everything” in the CCB archive may indicate increased public awareness of life-sustaining measures from medical and non-medical literature [2, 3], publicized legal cases [4, 5], and over-representation of success of life-sustaining measures portrayed in mainstream media [10]. The COVID-19 pandemic has further highlighted the need for thoughtful discussion surrounding end-of-life care for patients who are unlikely to survive despite maximal ventilatory support as well as patients denied of critical care due to resource shortages [41].

The main limitation of this study is that it furnishes only an indirect understanding of “do everything” since we are analyzing transcripts of testimony to the CCB, and not specific conversations between physicians and patients or SDMs. We acknowledge that since these were extreme cases that required adjudication by the CCB, they may not represent the typical local processes of goals of care discussions between physicians and SDMs, which generally end with consensus. Because findings of the CCB can be appealed, the absolute final verdict or outcome of each decision has not been explored and is outside the scope of this study. Our analysis was based on cases that appeared in the CCB archive as of 2018 and therefore we cannot speak to cases that may have occurred since then.

## Conclusion

This work points to the complexities of physician–patient and physician-SDM communication in the care of the critically ill, and the importance of clarifying whether one’s appeal to “do everything” has as its goal the patient’s quality of life or quantity of life. This divergence in perspectives on death versus suffering was consistently the locus of conflict. Clarity and transparency are to be valued, and potentially ambiguous phrases such as “do everything” should be avoided. To better understand the origins and limitations of “do everything” and similar utterances, future studies could seek to characterize and analyze such rhetorical phrases using a historical or philosophical lens.

## Data Availability

The datasets used and/or analyzed during the current study are available from the corresponding author on reasonable request.

## Abbreviations

SDM: Substitute Decision-Makers
CCB: Consent and Capacity Board
HCCA: Health Care Consent Act
CanLII: Canadian Legal Information Institute database
SICG: Serious Illness Conversation Guide
